# Materials Discovery With Machine Learning and Knowledge Discovery

**DOI:** 10.3389/fchem.2022.930369

**Published:** 2022-07-07

**Authors:** Osvaldo N. Oliveira, Maria Cristina F. Oliveira

**Affiliations:** ^1^ Sao Carlos Institute of Physics (IFSC), University of Sao Paulo, Sao Paulo, Brazil; ^2^ Institute of Mathematics and Computer Science (ICMC), University of Sao Paulo, Sao Paulo, Brazil

**Keywords:** materials discovery, machine learning, computational chemistry, knowledge discovery, data analytics

## Abstract

Machine learning and other artificial intelligence methods are gaining increasing prominence in chemistry and materials sciences, especially for materials design and discovery, and in data analysis of results generated by sensors and biosensors. In this paper, we present a perspective on this current use of machine learning, and discuss the prospects of the future impact of extending the use of machine learning to encompass knowledge discovery as an essential step towards a new paradigm of machine-generated knowledge. The reasons why results so far have been limited are given with a discussion of the limitations of machine learning in tasks requiring interpretation. Also discussed is the need to adapt the training of students and scientists in chemistry and materials sciences, to better explore the potential of artificial intelligence capabilities.

## Introduction

The ongoing revolution with Artificial Intelligence (AI) is certain to affect science and technology, especially in materials science, chemistry, and engineering owing to their interdependence. On one hand, the “hardware” component of intelligent systems, e.g., the sensors and actuators used to build soft robots and implement Internet-of-Things applications, will increasingly rely on advanced materials. Therefore, fulfilling the promises of AI requires developments in chemistry and materials sciences. On the other hand, machine learning and other AI methods have become crucial for materials design and discovery, in addition to their importance for treating the data generated from materials characterization (Rodrigues et al., 2021). AI comprises multiple and related research fields, including ML, natural language processing (NLP), computer vision, pattern recognition, robotics, and knowledge management. Hence, AI as a discipline spans a lot more than ML, a fact not always properly emphasized in communications targeted at non-experts or general audiences. This is possibly a consequence of ML being by far the AI methodology most deployed in diverse application areas, including chemistry, and materials sciences.

The main aim in this Perspective paper is to introduce knowledge discovery as a potential tool for fields of chemistry and materials sciences. In order to do that we provide some context information illustrating the current uses of ML in solving problems in chemistry and materials, but with no intention of providing a comprehensive account of such uses, as this is not a Review paper on the topic. We do place considerable emphasis on the strengths and limitations of present ML methodologies. In particular, we explain why ML is highly efficient for solving tasks that rely on learning by recognizing patterns, but not so for handling tasks that require cognition and interpretation. Since this is crucial for understanding the potential of extending ML to handle knowledge discovery tasks, we provide some background information on computer science and NLP to clarify the limitations identified. In an outlook section we present some predictions for the short-term future in the field, which include topics associated with knowledge discovery. The increasing usage of ML and AI also has implications for the training of students and scientists, which we discuss briefly. We do not develop the distinct but related topic of novel materials to produce AI devices, which is mentioned in the Concluding Remarks, along with some prospects of performing ML *via* hardware.

## Machine Learning Applied to Chemistry of Materials

**FIGURE 1:**
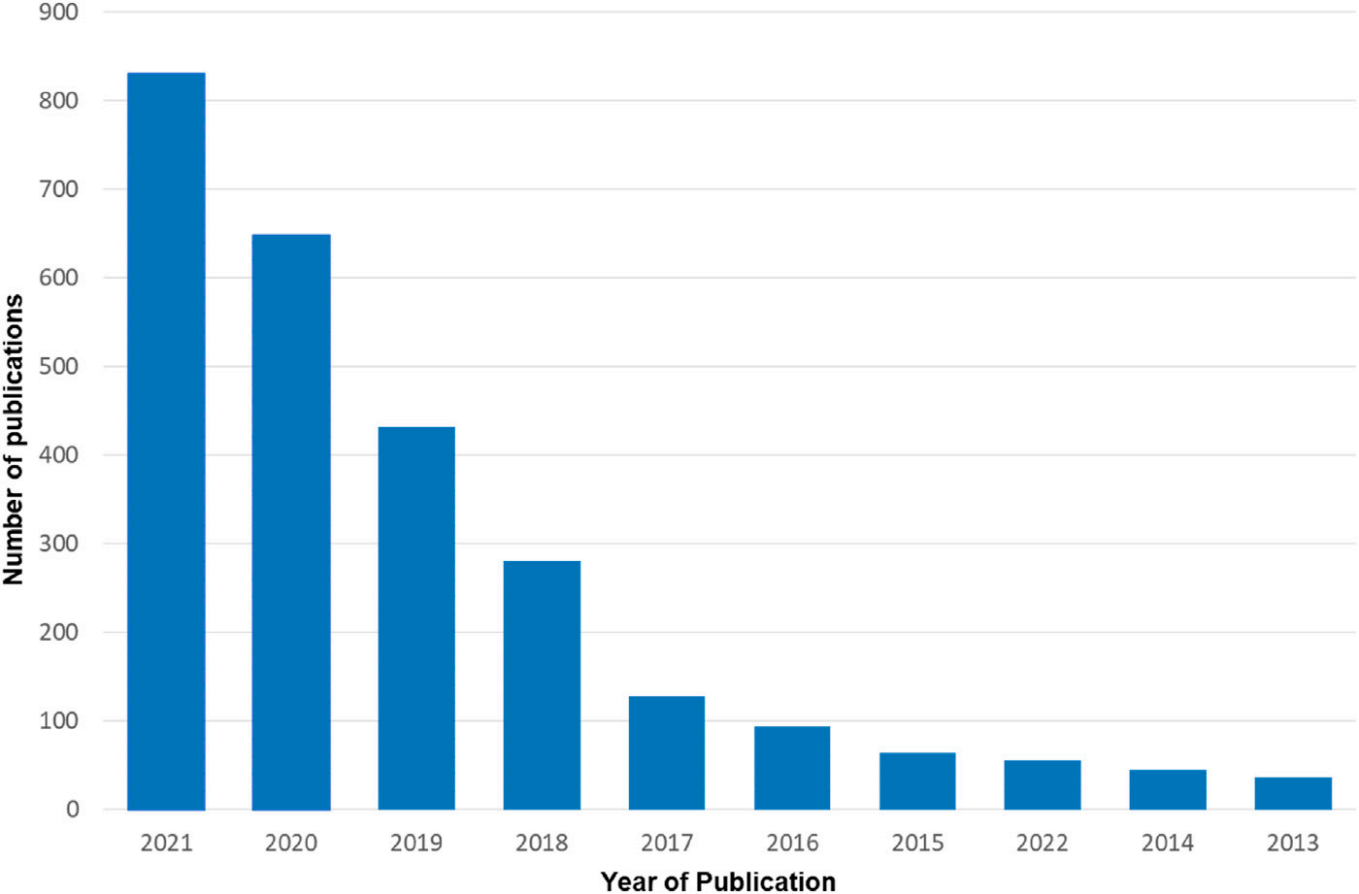
Papers retrieved from the Web of Science using the query “machine learning and (chemistry or materials discovery)” on 21 January 2022, per publication year.

The connection between chemistry, materials science or nanotechnology with computational methods has been discussed in review papers (Oliveira et al., 2014; Rodrigues et al., 2016; Paulovich et al., 2018), with examples of applications in many research areas [see (Paulovich et al., 2018; Rodrigues et al., 2021) for an overview]. Of special relevance in this context is the area of materials design and discovery, today heavily based on ML methods. Just by way of illustration, a query in the Web of Science in January 2022 with the keyterms “machine learning and (chemistry or materials discovery)” retrieved over 2,800 papers, of which more than half published in the last 2 years, as indicated in Figure 1. As far as the output in scientific papers is concerned, the field has been dominated by the United States, with almost 1,200 publications, and Europe, with nearly 1,000 publications. The appearance of AI and ML as relevant keywords in chemistry and materials sciences literature is relatively recent, and one may expect a growth in this field even higher than the one observed so far.

One can broadly identify three major types of ML applications for chemistry and materials sciences, as follows:

1) Data analysis. As huge amounts of data are generated from increasingly sophisticated equipment and computer simulations, researchers will inevitably rely on computational methods to process their data. This is true even in scenarios where data are acquired locally by individual researchers and the amount of data to be processed is small, far from being categorized as Big Data. The most significant example is in sensing, biosensing, and diagnostics, of any type (Paulovich et al., 2018; Rodrigues et al., 2021).
2) Materials design and discovery. This area has gained momentum in recent years owing to the Materials Genome initiatives [see comments in (Rodrigues et al., 2021)] and availability of high-throughput experiments and simulations to build libraries of materials properties. Coupled with the dissemination of robust implementations of established ML algorithms, these initiatives have fostered data intensive approaches for drug discovery and materials design.
3) Knowledge discovery. In computer science, knowledge discovery typically refers to the process of using ML algorithms to extract useful and actionable information from data (Fayyad et al., 1996). In this article we are concerned with the extraction of useful information from text, more specifically scientific literature, for purposes of mapping the existing knowledge on one or multiple topics of interest. The term “knowledge discovery” is rather novel for the chemistry and materials sciences communities, but we consider it here owing to its anticipated potential impact in not-too-distant a future. Indeed, recent contributions (Silva et al., 2016; Kim et al., 2017; Extance, 2018; Kogonova et al., 2021) have shown that automated (or semi-automated) tools may soon become available for researchers to extract and organize information from the literature, which may go beyond academic publications.

The analysis of scientific data, from sensors and other devices, for monitoring and diagnosis has long relied on ML algorithms, particularly for those cases in which pattern recognition methods are the most suitable. Prototypical examples are in the use of electronic tongues to assess the quality of wine and coffee (Riul et al., 2004), to identify opiate illicit drugs (Ortiz-Aguayo et al., 2022), and to diagnose oral cancer (Braz et al., 2022). In the latter paper, impedance spectroscopy data obtained with an electronic tongue applied to saliva samples were combined with clinical information to enhance diagnosis accuracy. Supervised machine learning provided a reasonable accuracy of 80% based on the data of less than 30 cancer patients and healthy individuals. Though the number of patients and healthy volunteers was insufficient to yield definitive conclusions, results indicated that alcoholism may be associated with oral cancer (Braz et al., 2022), a relevant finding not anticipated by the medical doctors involved in the study. This finding is representative of the potential of combining distinct types of information in diagnosis, a major advantage afforded by machine learning approaches.

Several review papers have discussed the landscape of materials discovery based on machine learning (Juan et al., 2021; Gao et al., 2022; Karthikeyan and Priyakumar, 2022; Wang et al., 2022), in some cases providing references to available databases (Gao et al., 2022). We mention a couple of examples by way of illustrating specific applications. Wang et al. (2022) identified ideal double perovskites for efficient photovoltaic devices with a ML workflow that screened all suitable double perovskites from the periodic table. The properties of these perovskites could be computed with a predictive accuracy of 92%, orders of magnitude faster than would be possible with *ab initio* calculations. More than 23,000 double perovskites not yet explored were screened, and six had adequate band gaps, from 1.0 to 2.0 eV, of which two have direct band gaps (Wang et al., 2022). A similar problem is encountered in trying to discover new two-dimensional (2D) materials. Existing databases already contain information on several thousand 2D materials, but the chemical design space is much vaster. A deep learning generative model made it possible to obtain over 267,000 new potential compositions for 2D materials (Song et al., 2021). While energy calculations serve to verify which of these materials can actually be formed, the approach using random forests could predict the crystal structures of 92 compositions in a subset of the hypothetical formulas. Significantly, their structure stability could be confirmed with density functional theory (DFT) calculations (Song et al., 2021). A review on the use of machine learning for materials discovery, with an emphasis on 2D materials, can be found elsewhere (Schleder et al., 2021).

## Knowledge Discovery and Natural Language Processing

Knowledge discovery in chemistry and materials sciences is yet at an embryonic stage as compared with application of ML in data analysis and materials discovery, as already mentioned. It depends heavily on NLP, a field that until recently faced severe practical limitations. Nonetheless, a few examples of knowledge discovery in materials science can be found in the literature, as in the analysis of over 12 thousand manuscripts to predict relevant parameters for the synthesis of titania nanotubes (Kim et al., 2017). ML and NLP techniques were combined to retrieve relevant articles automatically and extract synthesis conditions and materials properties from the text, thus permitting to discover the relationships between synthesis conditions and materials. Synthesis steps such as hydrothermal and calcinations reactions could be recognized, but authors highlight that the ML models could not distinguish multiple synthesis routes appearing in a single paper (Kim et al., 2017). This limitation is indicative of the difficulties faced in interpreting text, even in a seemingly simple scenario in which all that was required was determining the text boundaries of different synthesis routes.

Another interesting example found was an analysis of solid-state chemistry literature to improve understanding of materials synthesis (He et al., 2020). A major difficulty in the analysis is to identify which materials are precursors and which are targets. Following the strategy of named-entity recognition in NLP, which basically consists in the automatic identification of entities in a text, He et al. (2020) introduced a chemically-driven named-entity recognition model to identify precursors and targets. Their two-step model was based on a bi-directional recurrent neural network architecture (Bi-LSTM) (Lample et al., 2016) which takes advantage of context information, i.e., it considers the entities identified as materials and their surrounding words. He et al. (2020) developed a predictive synthesis model with a knowledge discovery effort that required data collection and preparation: they compiled over four million papers from major publishers, and employed a semi-supervised random forest model to identify 371,850 paragraphs describing inorganic synthesis of various types. From these, 95,283 paragraphs and their corresponding abstracts from 86,554 papers were used for materials extraction.

## Artificial Intelligence, Knowledge Discovery and Integrated Platforms for Materials Research

**FIGURE 2:**
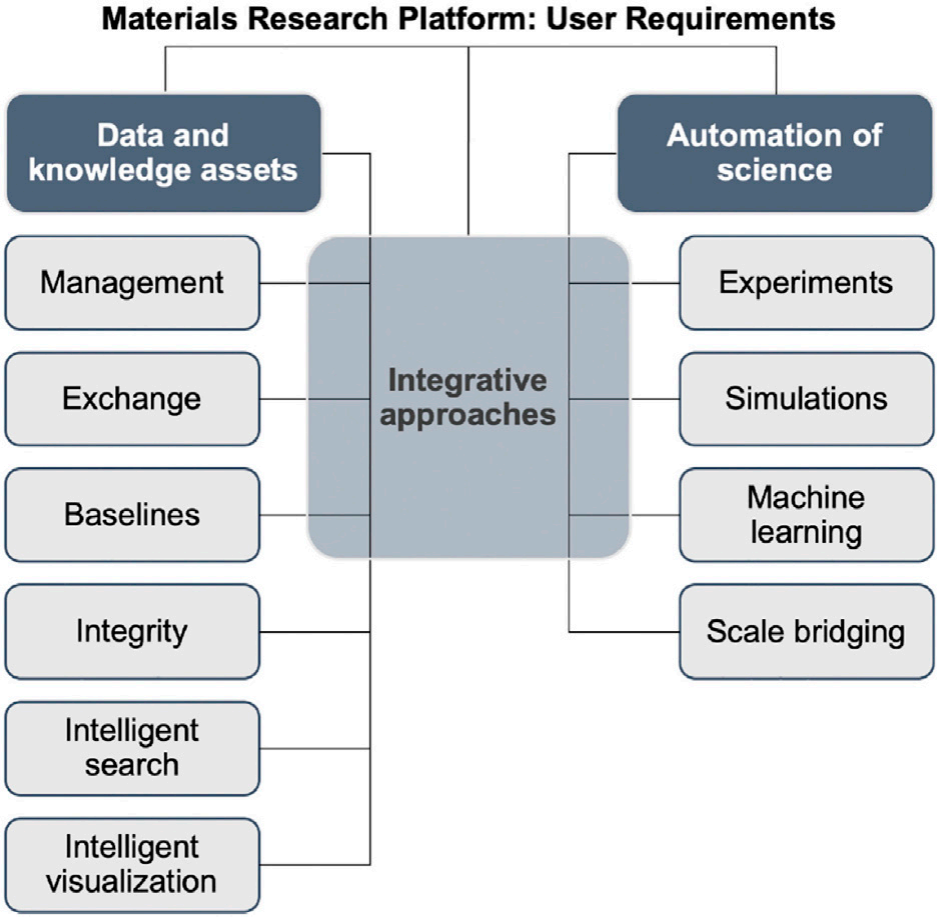
Requirements considered as the Main Pillars of a Materials Research Platform, within three broad themes: Data and Knowledge Assets, Automation of Science on the Platform, and Integrative Approaches. Reproduced from ref. (Aykol et al., 2019).

Strong support for the integration of chemistry, materials science, and engineering with AI methodologies came from a proposal of a Materials Research Platform, produced by a team of 40 experts in various areas (Aykol et al., 2019). These experts identified the following 16 requirements for a useful platform: Adaptive systems—active-learning and beyond; Automation of experiments; Automation of simulations; Collaboration; Data ingestion and sharing; Integration; Knowledge discovery; Machine learning for experiments; Machine learning for simulations; Multi-fidelity and uncertainty quantification; Reproducibility and provenance; Scale bridging; Simulation tools; Software infrastructure; Text mining and natural language processing; Visualization. The requirements have been conceptually organised into three interrelated themes, which are illustrated in Figure 2: 1) data and knowledge assets, 2) automation of science, and 3) integrative approaches. Despite the varied nomenclature, one may fit these requirements and themes within two big movements, viz. Big Data and NLP. Within the framework of the Big Data movement, they are basically related to the methods for acquiring, curating and analysing data (experimental, theoretical, and simulations), whereas strategies for knowledge management and intelligent search are typical of NLP.

Perhaps the most unforeseen requirements in Figure 2 are associated with knowledge management and the so-called baselines [“baselines help gauge where a new scientific finding stands but are often lacking” (Aykol et al., 2019)]. We consider these concepts and associated methodologies as being strongly related to leveraging the existing literature in a field. In fact, the study of the literature is an integral part of any research project, and the large number of papers and patents in any given field poses a major challenge to this task. Researchers may have to consult or survey hundreds if not thousands of items in the literature, which can no longer be done manually in a reasonable timeframe. Semi-automated tools (Silva et al., 2016; Extance, 2018) have been devised to assist in analysing content retrieved for surveys, including approaches specific for materials-related topics (Kim et al., 2017; Kogonova et al., 2021). The importance of these requirements justifies a stronger connection between NLP and materials science and engineering and further attention to the topic of knowledge discovery from text. A search on Chemistry of Materials on 24th January 2022 for the keyterm “natural language processing” has retrieved only three entries. “Knowledge Discovery” has not appeared yet, though there are odd appearances in ACS Nano, ACS Applied Materials and Interfaces and the Journal of the American Chemical Society (one entry in each journal). Our motivation for discussing knowledge discovery in this perspective paper lies in its tremendous potential enabled by recent developments on NLP associated with ML, despite acknowledging the modest results so far.

**FIGURE 3:**
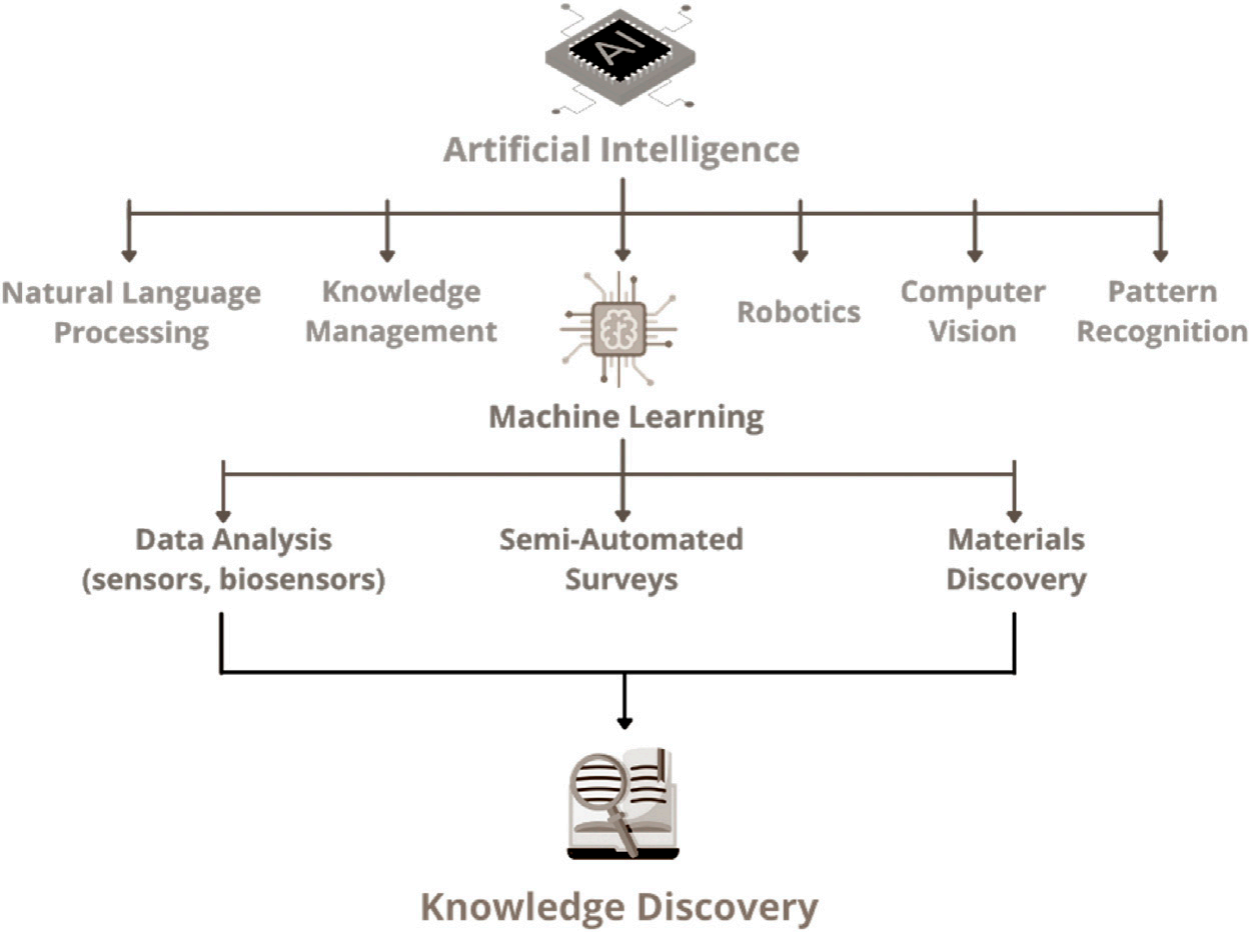
Block diagram illustrating the main AI methodologies, emphasizing the potential applicability of ML in data analysis, materials discovery, and semi-automated surveys. Knowledge discovery is placed as the ultimate AI application, which would be essential for the new paradigm of machine-generated knowledge.

The practical results of knowledge discovery [as the ones illustrated in refs. (Silva et al., 2016; Kim et al., 2017; Kogonova et al., 2021)] have been limited, especially owing to the challenges associated with automated text interpretation by machines. Yet, it is evident that AI software tools will likely assist humans in any literature survey effort in the near future. Publishers are already developing AI tools to assist editors, and authors may soon benefit from analogous tools, which makes NLP a key area for science and technology. In fact, a broader perspective of the impact and breadth of AI may be inferred from recent predictions (Oliveira and Oliveira, 2021; Rodrigues et al., 2021) that in a few decades the machines themselves may be able to generate knowledge. This new paradigm, with machines capable of generating knowledge without human intervention for the first time in history, will reshape society. The block diagram depicted in Figure 3 shows multiple AI methodologies, NLP included, and places knowledge discovery as the output of AI processes, which as far as chemistry of materials is concerned is mostly based on ML. Nonetheless, the next significant moves in the field will probably rely on the integration of ML with other AI methodologies. Indeed, even though NLP and ML appear in the figure as distinct subareas of AI, their boundaries are not clearly defined and they are actually combined in many applications, as it will be commented upon later on.

## Machine Generated Knowledge

Reaching the ambitious goal of machine-generated knowledge will likely result from the convergence of two major movements already pointed out in this text: the Big Data movement through ML, which is critical for data intensive discovery, and the NLP movement, which is critical for knowledge discovery. Both movements are intertwined, as in order to generate knowledge machines must learn sufficient information about the world. This is only possible if they are capable of processing text and natural language effectively, as humans do. As the two movements are already underway, one may wonder why machine-generated knowledge has not yet become a reality. A precise answer to this question must consider the current limitations of AI methodologies, and of ML in particular.

## Limitations of ML

Machine learning handles two broad classes of problems, namely those that require identifying relevant patterns and those that require interpretation. A typical task of the first type is classification, and abundant examples of successful classification tasks are seen in image analysis and facial recognition applications. Provided there is sufficient data to train the algorithms and sufficient processing power, intelligent systems for classification normally surpass the human ability. Yet, AI does not replace expert knowledge. For a start, classification typically relies on pattern identification, but it is not always possible for a human expert to observe and understand the reasons that led an algorithm to output a certain result. In many cases obtaining a correct classification is not sufficient and some level of explanation of the result should also be provided, e.g., in clinical diagnosis. Current ML algorithms focused on pattern identification simply ignore any underlying cause-and-effect relations, which are nonetheless central to explaining observed behaviours in the real world. It should be noted, nevertheless, that though at its very core ML is always based on pattern identification, there are approaches that allow identifying rules explaining cause-and-effect relations, as in the concept of multidimensional calibration space, further discussed in the Outlook section.

The same efficacy observed in classification tasks does not apply to interpretation tasks executed by ML algorithms. For tasks that require answering questions of the type “How?” “Why,” i.e., demand some degree of understanding and interpretation, the success of intelligent systems is much narrower. In fact, this limitation explains why NLP tools have so far failed to provide interpretation of texts or high-level communication between humans and machines. This brings us to a discussion on the nature of current capabilities and limitations of ML.

Major breakthroughs in ML happened with recent implementations of the connectionist model of AI on deep neural network architectures, yielding the so-called deep learning (DL) algorithms. Several algorithms and architectures have been successful in executing “difficult” tasks of an “intellectual” nature, such as language translation, object or face recognition, and speech to text conversion. As they are currently approached, these can typically be framed as classification tasks. In general, DL and its underlying technology, deep neural networks, are deemed suitable for tasks that require efficient detection of representative patterns in large data sets. In materials science, for instance, DL with decision tree algorithms has been used to generate high dimensional potential energy models for 54 elemental systems and alloys (Manna et al., 2022). Algorithms learn models from large sets of annotated data (the “training data”), from which they can extract and learn the relation between the data patterns and the output classes. Thus, as of now, successful ML requires humans to provide the high-level concepts in the form of labels, an approach known as “supervised learning”.

In his seminal work, Kahneman (2011) identifies two types of cognitive processes in humans. The “system 1 cognition” refers to unconscious, low level and fast cognitive processes that require no effort, limited attention and no explicit reasoning. Tasks solved with system 1 cognition involve intuition and habit. The “system 2 cognition” refers to conscious, high level, and typically slow cognitive processes that involve reasoning, logic, inference and explanation—closer to our idea of “interpretation” tasks. At present, DL algorithms are good at solving tasks typical of “system 1” cognition (Kahneman, 2011). There are many discussions on the limits of DL to handle higher level tasks of the “system 2” type, and thus getting closer to creating systems with learning performance more akin to the human capabilities when solving problems. While some authors advocate human-level AI requires completely novel paradigms, the pioneers Bengio, Lecun and Hinton (Bengio et al., 2021) argue that DL architectures can learn higher-level cognitive tasks if extended with some capabilities inspired on human learning. First, humans can generalize from rather limited experience, whereas current algorithms that use supervised learning require too much labeled data. Here, much progress has been observed in the field of transfer learning, in which the parameters learned by an architecture are transferred to a novel architecture that will solve a similar, yet different task (an example for materials science is introduced in the next section), which reduces the required training effort. Second, while humans need few examples to adapt to changes in the world, current DL algorithms are not robust to changes in data distribution. Actually, ML algorithms typically assume that the test cases come from the same distribution as the training cases, which is unrealistic for data collected in the real word. Third, they point DL architectures should evolve to handle more complex tasks, e.g., requiring a deliberate sequence of steps. For instance, architectures could incorporate attention mechanisms to focus on the bits of information relevant at a particular processing stage, rather than in the whole input information (Bengio et al., 2021), a concept already in use in recent DL architectures for NLP tasks.

## Outlook

The upcoming revolution in science and technology yielded by the new paradigm based on machine-generated knowledge will only be achieved after overcoming the current limitations in AI and ML. As for now, it is not possible to make a firm prediction of when this is going to happen. Yet, analysing short-term perspectives may be of practical value. We anticipate an increasing usage of ML in data analysis in the field of chemistry and materials sciences, particularly in diagnosis and monitoring based on results from sensors, biosensors, images, and videos. Since any diagnostics is *per se* a classification task (even when regression is employed), the use of ML will be a must. For instance, the construct related to analytical curves has been extended with machine learning in the new concept referred to as multidimensional calibration space (Popolin-Neto et al., 2021). With this concept, one can not only predict but also explain how samples are classified in a diagnosis exercise, with rules that resemble (and replace) the equations used in calibration curves. Also significant is the overall principle according to which, for ML algorithms, the data type or source is irrelevant for the task to be performed. In other words, “Data is data”. This principle could appear intriguing to a chemist or a materials scientist, because the common knowledge is that the nature of the information conveyed and the expertise required to analyze data will vary considerably in distinct problems. This is not so for an intelligent system that relies on a classification algorithm, since as long as the input features and the classes are well defined, the classification task will be performed regardless of the specificities of the data instances. An implication of this principle is the generality of ML algorithms, which may be employed in tasks as diverse as clinical diagnosis with image processing, sample classification with electronic tongues and noses, and even text classification (Oliveira et al., 2014; Rodrigues et al., 2016; Paulovich et al., 2018; Oliveira and Oliveira, 2021; Rodrigues et al., 2021).

In the discovery of new materials and new properties, successful use of ML will require curation of large materials properties databases (Oliveira et al., 2021), probably with initiatives similar to those of the materials genomics projects (Breneman et al., 2013). Furthermore, researchers need to be aware of the strengths and limitations of existing approaches. While several ML tasks are employed in addressing chemistry problems, at present viable solutions to scientific-technological problems can only be obtained using algorithms that rely on pattern identification. Also relevant is to consider the critical amount of data required for successful application of some ML algorithms. Deep learning strategies, for instance, require comprehensive datasets, unless transfer learning can be applied with datasets from other domains. Transfer learning has been advocated as suitable to predicting materials properties because chemical, electronic, thermodynamic, physical, and mechanical properties are interrelated (Yamada et al., 2019). The rationale behind this strategy is that, when there is only a limited supply of training data for a given target property, pretraining is made in models of related proxy properties for which there is sufficient data. For instance, using a pretrained model library referred to as XenonPy.MDL, comprising over 140,000 pretrained models for small molecules, polymers, and inorganic crystalline materials, Yamada et al. (2019) built models departing from only dozens of materials data, also revealing underlying connections between small molecules and polymers. In one of the applications, a prediction model was obtained which described the specific heat capacity of polymers as a function of chemical structures of the repeat units (Yamada et al., 2019).

The need of large databases is one of the stringent requirements for exploiting ML and AI. In fact, in proposing a materials platform Aykol et al. (2019) emphasize that integrating consortia of research groups and institutions is necessary to provide the required expertise and cope with the sheer size of the databases already available. In their own words (Aykol et al., 2019): “Materials science of the future is expected to be interwoven with data, automation, machine learning, and other emerging information technologies.” Integrated data curation, sharing and dissemination are already observed in commercial and academic platforms, e.g., two examples are MaterialsZone (https://www.materials.zone/) and Materials Cloud (Talirz et al., 2020). The emergence of data integration platforms is also driven by a growing concern with data generation and usage adherent to the FAIR data principles, namely Findability, Accessibility, Interoperability, and Reusability (Wilkinson et al., 2016). The principles of open science are critical to accelerating future progress, as open publication of datasets, source codes and neural architectures can contribute to overcoming many practical limitations in adopting novel research practices and workflows.

The discussion in this paper concentrated on supervised machine learning, currently the most employed paradigm in materials discovery. However, examples may also be found which use unsupervised machine learning, and considerable progress may be anticipated with methods exploiting variational autoencoders (Kingma et al., 2016). As an example in chemistry and materials, deep generative models were obtained for designing nanoporous crystalline reticular materials using a variational autoencoder (Yao et al., 2021).

In a future in which AI methodologies play such a critical role in research, it is also relevant to consider the impacts in education and training of researchers. For beyond the concepts and skills associated with the (already) many topics in materials, other skills will be required from students expected to work in a scenario dominated by large research consortia. These skills include the mastering of fundamentals of information technology, artificial intelligence and data science, and the ability to work in teams. Therefore, graduate programs in chemistry and materials science will need to enhance their inter- and multidisciplinary content and focus, and the teams of supervisors and researchers will have to be extended beyond the borders of chemistry and materials science. Those programs more likely to be successful will prioritize increased diversity—of areas, backgrounds and skills—including those not directly related to materials, such as linguistic and social skills. There are at least three levels for such training, depending on the type of activity intended.

1) Students could be trained to interact with AI experts, learning to identify opportunities in applying AI to their research. They would not need to learn how to write computer programs and not even use the software packages implementing ML algorithms. However, they should understand the concepts, strengths, limitations, risks of misuse, and the machinery behind the software packages.
2) Students could be trained to use the software packages implementing the ML algorithms, in addition to the skills mentioned in 1).
3) Students could be further trained to use the software packages and write programs implementing ML algorithms and other AI programs.

## Concluding Remarks

In this paper we have highlighted ML applications to solve problems of materials and their applications, but the opposite movement is also equally important, i.e., how materials sciences can yield devices that will enable novel AI applications. There is a long list of such materials and devices (Oliveira et al., 2021), including wearable sensors and actuators for virtual reality environments and Internet-of-Things applications, soft materials for flexible electronics and soft robots. In health and in the assessment of athletes’ performance, intelligent monitoring systems will require functional materials for wearable and even implantable biosensors. Similarly, intelligent systems for artificial vision and mimicking the human senses are all based on nanomaterials, as exemplified with electronic tongues, electronic noses, electronic skins (Paulovich et al., 2018; Oliveira and Oliveira, 2021; Rodrigues et al., 2021). In fact, there is reciprocity in contributions in all of these systems, for research on materials is essential to produce the devices, and ML (or another AI method) is used to analyze the data generated by such devices and make decisions. Emblematic examples of the latter are speech processing and voice recognition systems (Solanki et al., 2017) where sound detection is performed with strain sensors in wearable devices.

Today all the ML applications in extensive use are based on software, as ML *via* hardware is still embryonic, and it may take decades before it is seen in practice. However, tremendous potential for innovation exists in research in materials sciences. An example is the development of organic electrochemical devices to mimic synapses for neuromorphic computing (Van de Burgt et al., 2017). In addition, in this paper we only considered methods with classical computing, even in the more far-fetching predictions of future developments. Obviously, when quantum computers become available to run ML algorithms and apply other AI methods, a whole host of new possibilities will emerge. The expected innovative capabilities of quantum computing are very likely to significant accelerate reaching machine-generated knowledge, even though it may still take some time for such a breakthrough to reach practical applications.

## Data Availability

The original contributions presented in the study are included in the article/supplementary material, further inquiries can be directed to the corresponding author.
